# Emotion Dysregulation in College Students: Contributions of Maladaptive Personality Traits and Momentary Affect

**DOI:** 10.1007/s10862-025-10243-7

**Published:** 2025-08-04

**Authors:** Ethan M. Weires, Leiana de la Paz, Brendan M. Whitney, Molly A. Nikolas

**Affiliations:** 1 Department of Psychiatry, Massachusetts General Hospital, Boston, MA USA; 2 Department of Psychiatry, Harvard Medical School, Boston, MA USA; 3 Department of Psychological and Brain Sciences, University of Iowa, Iowa City, IA USA

**Keywords:** Emotion Dysregulation, Personality, Affect, Momentary

## Abstract

Emotion regulation involves behavioral modulation of emotional expression across contexts and is highly relevant to the experience of psychopathology. Maladaptive personality traits may be particularly relevant individual differences for understanding problems with emotion regulation, or dysregulation. Similarly, momentary variability in negative affect, which can be captured with greater precision via ecological momentary assessment (EMA) methods, may also be relevant for mapping how individual differences influence emotion dysregulation in real time. The present study investigated how emotion dysregulation is affected by maladaptive personality and negative affect among college students. Data were collected between September 2019 and September 2021, as part of a larger study on emotional functioning and social media use. Of the full baseline sample (*N* = 406), 154 students (*M*_age_=18.7, 72.7% female, 80.5% White) completed 28 EMA surveys over seven days. Path analysis revealed that all maladaptive personality traits, except antagonism, uniquely predicted multiple dimensions of dysregulation, with negative affectivity observed as the strongest trait predictor. Multilevel models demonstrated that both increased between- and within-person variability in negative affect were associated with increased overall levels of momentary dysregulation. Further, increased fluctuations in negative affect were associated with greater fluctuations in momentary dysregulation. These associations were significantly moderated by higher mean-levels of negative affect but not by any of the maladaptive traits. This study’s findings highlight notable differences in state and trait level predictors of dysregulation and contribute to the understanding of how such antecedents could be used in the development of real-time interventions.

**Supplementary Information:**

The online version contains supplementary material available at 10.1007/s10862-025-10243-7.

## Introduction

Emotion regulation is defined as the ability to influence the trajectory and expression of one’s emotions (Gross, 2015) and is a frequently targeted transdiagnostic process relevant to the onset and maintenance of psychopathology (Gross & Jazaieri, 2014; Sheppes et al., 2015; Werner & Gross, 2010). Individuals with difficulties successfully employing emotion regulation (i.e., those experiencing emotion *dysregulation*) are at higher risk for psychopathology (Aldao et al., 2010; Cole et al., 1994), a link that is explained, at least in part, by opposite, problematic responses to emotion (e.g., rumination, avoidance; Aldao et al., 2010; Ford et al., 2014). There are significant individual differences at play in the employment of regulatory processes and in the use of adaptive versus maladaptive strategies for regulation (Garnefski et al., 2002; Naragon-Gainey et al., 2017; Webb et al., 2012).

Personality traits derived from the Five Factor Model of Personality (FFM; McCrae & John, 1992) are a set of individual factors that appear to account for differences in emotion regulation (Barańczuk, 2019; Eldesouky & English, 2019). High levels of conscientiousness have been linked to lower negative emotion reactivity (Komulainen et al., 2014) as well as the more frequent usage of adaptive regulatory strategies like problem-solving (Barańczuk, 2019; Southward et al., 2018). Individuals high in agreeableness tend to exhibit a heightened sensitivity to the stress of others (Suls et al., 1998), a reduced sensitivity to their own daily stresses (Komulainen et al., 2014), and report that their primary intention for regulation is to avoid worsening the affect of others (López-Pérez et al., 2019). Individuals high in extraversion primarily demonstrate pro-hedonic goals (Tamir, 2009), utilize distraction, acceptance, and reappraisal (Barańczuk, 2019), and generally employ more adaptive and proactive strategies (López-Pérez et al., 2019; Southward et al., 2018). Particularly relevant to clinical aims are the associations identified between neuroticism, emotion dysregulation, and the use of maladaptive regulation strategies (Cheshure et al., 2020; Pollock et al., 2016; Yang et al., 2020).

Emotion dysregulation has been implicated in psychopathology across its stages from etiology to its maintenance and consequences (Berking & Wupperman, 2012; Lincoln et al., 2022). Particular attention has been dedicated to characterizing its role in personality pathology, with the Diagnostic and Statistical Manual of Mental Disorders, Fifth Edition, Text Revision (DSM-5-TR; American Psychiatric Association, 2022) noting difficulties with affectivity as one of the four main areas of general personality disorder. Notably, emotion dysregulation is considered to be a core feature of borderline personality disorder (BPD; Bud et al., 2023; Carpenter & Trull, 2013; Chapman, 2019; Linehan, 1993). Efforts to study dysregulation in these contexts have greatly strengthened the field’s understanding of how it contributes to personality pathology but may fall short of determining the associations between dysregulation and pathological features of personality beyond diagnoses. Use of dimensional measures of personality pathology, such as the Personality Inventory for DSM-5 (PID-5; Krueger et al., 2012) may be helpful for understanding the role of individual trait differences in different aspects of emotion dysregulation.

The assessment of dysregulation on a momentary timescale has also helped to facilitate the identification of individual factors that contribute to emotion dysregulation. Emotion regulation processes are highly dynamic, underscoring the potential role that momentary and situational factors play in the unfolding of these processes (Aldao, 2013). There has been a recent surge in the use of ecological momentary assessment (EMA) methods to better capture the dynamic nature of these regulation processes (Colombo et al., 2020; Koval et al., 2023). EMA studies have addressed differences in the implementation of regulation strategies (Alawadhi et al., 2023), the linkage of specific emotions to regulation strategies (Smith et al., 2023), and the relation of individual factors to disengagement (Sandel-Fernandez et al., 2023). Through analyses of within-person differences in emotion, findings emphasize the relevance of both intensity and variability in negative emotionality to challenges with regulation (Aldao et al., 2015; Blanke et al., 2020; McMahon & Naragon-Gainey, 2019; Smith et al., 2023). Further, these momentary effects may also be moderated by individual trait-level factors (Sandel-Fernandez et al., 2023); however, to our knowledge, no prior work has specifically evaluated maladaptive personality traits as potential moderators.

The purpose of the current study was to extend the knowledge in these areas of personality pathology and momentary emotion regulation by first examining the unique associations between maladaptive personality traits and dimensions of emotion dysregulation. This study further examined the contributions of momentary negative affect to the prediction of both between- and within-person variability in dysregulation. Finally, we evaluated whether maladaptive personality traits moderated associations between momentary negative affect and dysregulation. For our first aim, we predicted that negative affectivity, detachment, antagonism, disinhibition, and psychoticism would each uniquely contribute to multiple dimensions of emotion regulation problems, in line with prior work (Pollock et al., 2016). We also predicted that negative affectivity would show the largest unique effects common to multiple dimensions of emotion dysregulation. In relation to our second aim, we hypothesized that increased negative affect would predict increased momentary ratings of emotion dysregulation. Additionally, we predicted that higher between-person (i.e., mean level) negative affect as well as higher within-person variability in negative affect would each uniquely predict increased between-person levels of emotion dysregulation over the course of an intensive longitudinal period. Further, we predicted that greater within-person variability in negative affect would predict greater *within-person variability* in emotion dysregulation. Finally, in relation to our third aim, given the strength of these dimensions in prior work, we hypothesized that negative affectivity, detachment, and antagonism would moderate the effects of within-person negative affect on momentary ratings of emotion dysregulation, such that the association would be stronger for individuals with higher overall trait levels of negative affectivity, detachment, and antagonism.

## Method

### Participants

Participants were 406 undergraduate students recruited from a large, Midwestern university. Eligibility criteria required that participants be at least 18 years-old, be able to read and speak English, and have normal (or corrected to normal) vision and hearing. Of the 406 participants who completed the cross-sectional portion of the study, 190 were invited to participate in seven days of EMA, during which we collected information about their online activity habits, emotional and behavioral symptomatology (e.g., ADHD and depression), and experiences of emotion dysregulation. Because the goals of the original study involved evaluating the impact of social media use on ADHD, depression symptoms, and emotion regulation, individuals were invited to participate in EMA if they reported symptoms of ADHD and/or depression that were above the 84th percentile (+ 1 standard deviation) on the Adult Self-Report scale of the Achenbach System of Empirically Based Assessment (Achenbach & Rescorla, 2003). Below, we report how we determined our sample size, all data exclusions, and all measures used in the study.

First, quality control measures were implemented with the EMA data prior to analyses. In line with recent work evaluating the quality of EMA ratings (Jaso et al., 2022), we examined survey response times and anchor responses to evaluate data quality and eliminate data due to potential careless or random responding. This work suggests that both fast response times (i.e., survey duration time less than one second per item) and assessments which include the endorsements of all items at one response anchor or another each independently reflect careless responding. Based on these indices, 221 measurement occasions were removed. Following this step, we restricted the intensive longitudinal sample to those participants who provided 10 or more valid measurement occasions across the duration of the study. This resulted in a final sample of *n* = 154 participants in the EMA portion of the study.

The full baseline sample consisted of *N* = 406 participants (*M*_age_=19.0 years, *SD*_age_=1.17), who were 70.9% female, 76.4% White, 9.6% Hispanic, 6.2% Asian/Asian-American, 3.7% multiracial, 3% Black/African-American, 0.5% Hawaiian Native/Pacific Islander, and 0.2% Middle Eastern. Across the full baseline sample, current gross annual income was reported as 21.7% at less than $50,000, 24.6% between $50,000 and $100,000, 21.4% between $100,000 and $150,000 and 31.8% at greater than $150,000 (note: participants were asked to include their parent’s income if they were still financially supported by their parents). One participant did not complete all measures and was therefore dropped from several analyses, for which *N* = 405.

The EMA sample consisted of *n* = 154 participants (*M*_age_=18.7 years, *SD*_age_=0.98), who were 72.7% female, 80.5% White, 8.4% Hispanic, 5.2% Asian/Asian-American, 3.2% multiracial, 1.3% Black/African-American, and 0.6% Hawaiian Native/Pacific Islander. For the EMA sample, current gross annual income was reported as 20.1% at less than $50,000, 28.5% between $50,000 and $100,000, 19.5% between $100,000 and $150,000, and 31.8% at greater than $150,000.

### Procedures

Data were collected between September 2019 and September 2021. Participants completed either an in-person or online series of questionnaires at baseline (procedure moved online due to the COVID-19 pandemic), assessing multiple aspects of emotion and behavior, as part of a larger study examining emotional and behavioral functioning, psychopathology, and social media use among college students. Before completing study procedures, informed consent, including to the usage of data for secondary analyses, was provided by all participants either in-person or electronically. In addition to the measures relevant to the present study (detailed below), participants completed ratings on measures for ADHD, depression, anxiety, emotional reactivity, social media use and addiction, fear of missing out, social connection, and susceptibility to peer influence. Participants who completed baseline surveys in-person also completed several cognitive measures, which were dropped from the online protocol due to the onset of the COVID-19 pandemic. Participants who were invited to complete the EMA portion of the study received surveys four times per day (9 a.m., 1 p.m., 5 p.m., 9 p.m.) for seven days and began receiving surveys within four days of their completion of baseline questionnaires. This design allowed for a total of 28 EMA measurements possible per participant. All procedures were approved by the local Institutional Review Board.

### Measures

This following section lists the measures used in the present study. For a full list of all measures obtained in the original study, see Appendix A of the Supplemental Materials.

#### Emotion Dysregulation

Emotion dysregulation was measured with the Difficulties in Emotion Regulation Scale (DERS; Gratz & Roemer, 2004). The DERS asks respondents to self-report the extent to which they agree or disagree with 36 statements on a scale of 1 (almost never) to 5 (almost always). This measure provides a total score of emotion dysregulation (i.e., “Total DERS”) with a range of 36 to 180, where higher scores indicate greater emotion dysregulation. The DERS also contains six subscales, with higher scores indicating greater difficulties in each dysregulation category: nonacceptance of emotional responses (six items, range = 6–30; e.g., *when I’m upset*,* I feel ashamed with myself for feeling that way*), difficulty engaging in goal-directed behavior (five items, range = 5–25; e.g., *when I’m upset*,* I have difficulty focusing on other things*), impulse control difficulties (six items, range = 6–30; e.g., *when I’m upset*,* I lose control over my behaviors*), lack of emotional awareness (six items, range = 6–30; e.g., *I pay attention to how I feel* [reverse-scored]), limited access to emotion regulation strategies (eight items, range = 8–40; e.g., *when I’m upset*,* it takes me a long time to feel better*), and lack of emotional clarity (five items, range = 5–25; e.g., *I have difficulty making sense out of my feelings*).

#### Momentary Emotion Dysregulation

For momentary measures of emotion dysregulation, participants rated five items that represented the strongest loadings from factor analytic work on the DERS in college students (Victor & Klonsky, 2016). The intensive longitudinal surveys included instructions for participants to rate each item based on how they were feeling in the present moment on a 5-point scale (not at all to constantly), with scores ranging from 5 to 25. The prompt on the EMA survey read, “*In this moment*,* how much are you experiencing the following: difficulties making sense of my feelings*,* difficulty controlling my behavior because I am upset*,* feeling confused about how I feel*,* difficulty doing work because I am upset*,* and feeling irritated with myself because I am upset.*”

#### Maladaptive Personality

Maladaptive personality was assessed with the Personality Inventory for DSM-5–Brief Form for adults (PID-5-BF; Krueger et al., 2012) during the baseline portion of the study. The PID-5-BF asks respondents to report the degree to which each of 25 statements accurately describe them on a scale from 0 (very false or often false) to 3 (very true or often true). This measure provides a total score of maladaptive personality with a range of 0 to 75, where higher scores indicate greater overall levels of personality pathology. The PID-5-BF also contains individual scores for five maladaptive domains, each of which is measured by five items and includes a total range of 0–15. These domains are negative affectivity (e.g., *I get emotional easily*,* often for very little reason*), detachment (e.g., *I often feel like nothing I do really matters*), antagonism (e.g., *it’s no big deal if I hurt other peoples’ feelings*), disinhibition (e.g., *I feel like I act totally on impulse*), and psychoticism (e.g., *I often have thoughts that make sense to me but that other people say are strange*).

#### Momentary Negative Affect

We measured momentary negative affect with items from the Positive Affect and Negative Affect Schedule (PANAS; Clark & Watson, 1994). There were 10 affective labels chosen and measured at each EMA survey. Five labels measured positive dimensions of affect (e.g., *determined*), and five represented negative dimensions (e.g., *nervous*). Respondents were asked to report the extent to which they had been feeling each of the emotions since the last survey, with each emotion measured on a scale from 0 (not at all) to 5 (extremely). For the purposes of the present study, we utilized only ratings of negative affect and summed their scores for a total score ranging from 0 to 25. Note that these authors refer to this measure as *negative affect*, to be distinguished from the PID-5 trait *negative affectivity*.

### Analytic Strategy

To address our first aim, we conducted analyses to determine the unique positive associations between the five PID-5 traits and the six DERS dimensions of emotion dysregulation. We conducted bivariate Pearson correlations to examine the magnitude of associations between the personality traits and dimensions of dysregulation. Next, we used a path analytic model to evaluate unique associations between maladaptive personality traits and dysregulation dimensions while controlling for their intercorrelations. Covariates in this model included participant sex, age, and race/ethnicity.

To address our second aim, we computed between- and within-person indices of momentary negative affect. In line with previous work partitioning between- and within-person momentary variability (Wright et al., 2015), we computed a person-specific mean for negative affect across all 28 EMA measurement occasions. We computed 28 timepoint-specific measures of within-person variability in negative affect by subtracting the mean score from the individual timepoint score. This created indices of between- and within-person negative affect that were not correlated with one another. These indices were used as Level 2 (between-person) predictors of mean levels of dysregulation in a series of conditional multilevel models. In these models, we first fit a means-only model that included estimates of emotion dysregulation, measured longitudinally over the 28 timepoints. Next, we added the computed between- and within-person indices of negative affect as fixed effect predictors of momentary emotion dysregulation. Third, we included within-person negative affect as a random effect at Level 1 (within-person variability in emotion dysregulation).

To address our third aim, we included each of the personality traits as cross-level moderators of the fixed effect of within-person negative affect predicting momentary dysregulation. As an exploratory analysis, we evaluated whether mean levels of momentary negative affect moderated the association between within-person negative affect and momentary emotion dysregulation.

### Transparency and Openness

Sample size determination was originally based on power analyses conducted for detecting effects of social media use on ADHD, depression, and emotional functioning. Given the range of small to medium effects reported in the literature, we selected a sample size powered to detect small effects (Cohen’s *d* = 0.20) after controlling for the effects of covariates. These calculations indicated a minimum sample size of *N* = 315 at baseline to detect small effects with covariates. Power for the EMA portion was also estimated at 0.90 for detecting medium effects with an anticipated intra-class correlation of 0.50 and an approximate prompt response rate of 75%, based on a projected enrollment of *n* = 150 (for the EMA portion). Post-hoc power curves (included on the linked preregistration page) for our EMA sample of *n* = 154 indicated that we were well-powered to detect medium effects (power > 0.90) but not small effects (power < 0.60), based on our response rate (*M* surveys completed = 24.3 per participant; 86.8% response rate).

This study’s design and hypotheses, excluding the one noted exploratory analysis, were preregistered after data had been collected but before analyses were undertaken; see 10.17605/OSF.IO/H4W5Z. Data were analyzed using *R* v4.2.2 (R Core Team, 2022) and the packages *nlme* (v3.1-160; Pinheiro et al., 2022), *psychometric* (v2.3; Fletcher, 2022), and *ggplot2* (v3.4.2; Wickham, 2016). The dataset is not publicly available due to restrictions on public availability without explicit consent by rules of the local IRB. At the time of data collection, we did not obtain specific consent for public sharing of participant data. Interested researchers can contact the PI to request permission to access any of the deidentified data.

## Results

Table 1 contains a summary of descriptive statistics. Bivariate correlations are presented in Table 2. There were significant positive associations among all personality traits and dimensions of emotion dysregulation. Negative affectivity was most strongly correlated with limited access to regulation strategies (*r* =.68, *p* <.001) and non-acceptance of emotions (*r* =.62, *p* <.001), detachment with lack of emotional clarity (*r* =.53, *p* <.001) and limited access to regulation strategies (*r* =.51, *p* <.001), and antagonism with impulse control difficulties (*r* =.33, *p* <.001). Disinhibition was most strongly correlated with difficulties engaging in goal-directed behaviors (*r* =.39, *p* <.001), and psychoticism with limited access to regulation strategies (*r* =.58, *p* <.001) and non-acceptance of emotions (*r* =.57, *p* <.001). Lack of emotional awareness was found to be the dimension of dysregulation most weakly correlated with maladaptive personality traits, including its associations with antagonism (*r* =.20, *p* <.05) and disinhibition (*r* =.19, *p* <.05).

**Table 1:** Descriptive statistics

	*N* or M	% or SD	Min	Max
*Full Sample (N = 406)*				
Age (years)	18.96	1.17		
Sex (Female)	288	70.90		
Race (White)	310	76.40		
*EMA Sample (n = 154)*				
Age (years)	18.71	0.98		
Sex (Female)	112	72.70		
Race (White)	124	80.50		
Surveys completed	24.34	3.98		
*Baseline*				
* PID-5-BF (N = 405)*				
Negative affectivity	11.29	3.66	5	20
Detachment	7.92	2.73	5	17
Antagonism	6.85	2.24	5	20
Disinhibition	7.20	2.42	5	16
Psychoticism	8.64	3.23	5	20
* DERS (N = 406)*				
Non-acceptance of emotions	13.83	6.35	6	30
Difficulties engaging	11.29	4.31	5	23
Impulse difficulties	9.80	3.89	6	29
Lack of awareness	13.99	4.82	6	26
Limited access	16.87	7.81	8	39
Lack of clarity	11.81	4.46	5	25
Total DERS	77.60	25.30	39	159
*EMA (n = 154)*				
Negative affect	6.82	1.79	5	14.63
Emotion dysregulation	6.59	2.20	5	16.71

EMA-assessed negative affect and emotion dysregulation were measured at all 28 timepoints, across which a “person-level mean” was calculated for each participant. The means and standard deviations listed for these variables above were found by calculating the mean of participants’ person-level means for negative affect and emotion dysregulation

**Table 2:** Bivariate pearson correlations

	1.	2.	3.	4.	5.	6.	7.	8.	9.	10.	11.
1. Negative affectivity	-										
2. Detachment	0.50***	-									
3. Antagonism	0.39***	0.44***	-								
4. Disinhibition	0.26**	0.37***	0.48***	-							
5. Psychoticism	0.57***	0.62***	0.53***	0.38***	-						
6. Non-acceptance	0.62***	0.48***	0.28***	0.24**	0.57***	-					
7. Difficulties engaging	0.46***	0.43***	0.25**	0.39***	0.54***	0.64***	-				
8. Impulse difficulties	0.53***	0.45***	0.33***	0.29***	0.50***	0.63***	0.68***	-			
9. Lack of awareness	0.31***	0.40***	0.20*	0.19*	0.30***	0.39***	0.24**	0.24**	-		
10. Limited access	0.68***	0.51***	0.28***	0.27***	0.58***	0.76***	0.73***	0.66***	0.33***	-	
11. Lack of clarity	0.52***	0.53***	0.25**	0.26**	0.50***	0.67***	0.49***	0.55***	0.56***	0.59***	-
12. Total DERS	0.68***	0.59***	0.33***	0.33***	0.64***	0.88***	0.79***	0.77***	0.57***	0.89***	0.80***

N = 405. *p <.05, **p <.01, ***p <.001

### Maladaptive Personality and Emotion Dysregulation

Table 3 contains a full list of the standardized beta weights and confidence intervals, and Table 4 contains a list of the changes in R^2^ for the path analyses. The path analytic model simultaneously examined all five maladaptive traits as predictors of each of the six dimensions of emotion dysregulation, while also accounting for their intercorrelations. *Non-acceptance of emotions* was uniquely predicted by negative affectivity (*β* = 0.44, [0.34, 0.54], *p* <.001), detachment (*β* = 0.15, [0.04, 0.25], *p* =.008), and psychoticism (*β* = 0.19, [0.08, 0.30], *p* <.001). Similarly, *difficulties in engaging in goal-directed behavior* were uniquely predicted by negative affectivity (*β* = 0.32, [0.22, 0.42], *p* <.001), disinhibition (*β* = 0.19, [0.09, 0.29], *p* <.001), and psychoticism (*β* = 0.25, [0.14, 0.36], *p* <.001). *Impulse control difficulties* were uniquely predicted by negative affectivity (*β* = 0.39, [0.29, 0.49], *p* <.001), detachment (*β* = 0.14, [0.03, 0.25], *p* =.013), and disinhibition (*β* = 0.12, [0.03, 0.22], *p* =.012). *Lack of emotional awareness* was significantly predicted by detachment (*β* = 0.28, [0.15, 0.41], *p* <.001) and psychoticism (*β* = 0.16, [0.03, 0.29], *p* =.019). *Limited access to emotion regulation strategies* was predicted by negative affectivity (*β* = 0.47, [0.38, 0.55], *p* <.001), detachment (*β* = 0.22, [0.12, 0.31], *p* <.001), and psychoticism (*β* = 0.18, [0.08, 0.28], *p* <.001). Lastly, *lack of emotional clarity* was uniquely predicted by negative affectivity (*β* = 0.26, [0.16, 0.37], *p* <.001), detachment (*β* = 0.19, [0.07, 0.31], *p* =.001), and psychoticism (*β* = 0.28, [0.16, 0.40], *p* <.001).

**Table 3:** Path analysis standardized Beta weights

	Non-acceptance		Difficulties engaging		Impulse difficulties		Lack of awareness		Limited access		Lack of clarity		Total DERS	
Negative affectivity	0.44***	(0.34, 0.54)	0.32***	(0.22, 0.42)	0.39***	(0.29, 0.49)	− 0.01	(−0.13, 0.10)	0.47***	(0.38, 0.55)	0.26***	(0.16, 0.37)	0.41***	(0.32, 0.49)
Detachment	0.15**	(0.04, 0.25)	0.07	(−0.04, 0.18)	0.14*	(0.03, 0.25)	0.28***	(0.15, 0.41)	0.22***	(0.12, 0.31)	0.19**	(0.07, 0.31)	0.23***	(0.14, 0.32)
Antagonism	− 0.07	(−0.17, 0.02)	− 0.08	(−0.18, 0.02)	0.08	(−0.02, 0.18)	− 0.03	(−0.15, 0.09)	− 0.07	(−0.16, 0.01)	− 0.10	(−0.21, 0.00)	− 0.06	(−0.15, 0.02)
Disinhibition	0.03	(−0.06, 0.13)	0.19***	(0.09, 0.29)	0.12*	(0.03, 0.22)	0.05	(−0.06, 0.16)	0.06	(−0.02, 0.15)	− 0.00	(−0.10, 0.10)	0.09*	(0.01, 0.17)
Psychoticism	0.19***	(0.08, 0.30)	0.25***	(0.14, 0.36)	0.07	(−0.04, 0.18)	0.16*	(0.03, 0.29)	0.18***	(0.08, 0.28)	0.28***	(0.16, 0.40)	0.24***	(0.14, 0.33)

*N* = 405. Confidence intervals (α = 0.95) included in parentheses. *p<.05, **p <.01, ***p <.001

**Table 4:** Path analysis changes in R^2^

	Non-acceptance	Difficulties engaging	Impulse difficulties	Lack of awareness	Limited access	Lack of clarity	Total DERS
Negative affectivity	0.12***	0.06***	0.09***	0.00	0.13***	0.04***	0.10***
Detachment	0.01**	0.00	0.01*	0.04***	0.02***	0.02**	0.03***
Antagonism	0.00	0.00	0.00	0.00	0.00	0.01	0.00
Disinhibition	0.00	0.02***	0.01*	0.00	0.00	0.00	0.01*
Psychoticism	0.02***	0.03***	0.00	0.01*	0.02***	0.04***	0.03***

*N*=405. *p<.05, ** *p*<.01, ***p<.001

**Fig. 1:**
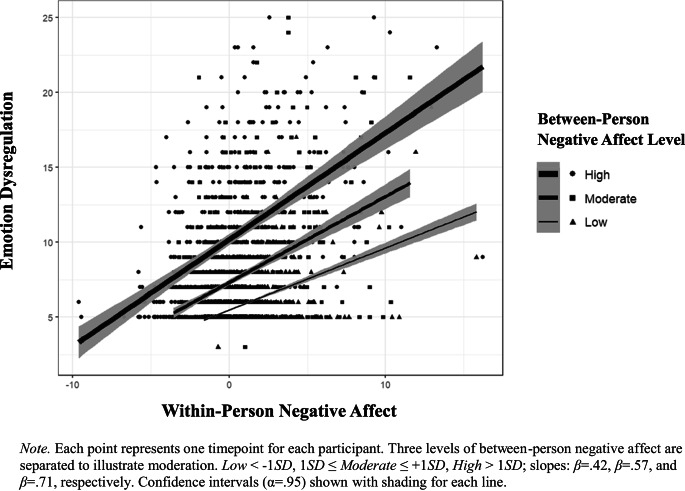
Moderating effect of between-person negative affect on emotion dysregulation

The same analyses were also performed for a total emotion dysregulation score, which was the sum of the six individual dimensions. *Total emotion dysregulation* was predicted by negative affectivity (*β* = 0.41, [0.32, 0.49], *p* <.001), detachment (*β* = 0.23, [0.14, 0.32], *p* <.001), disinhibition (*β* = 0.09, [0.01, 0.17], *p* =.037), and psychoticism (*β* = 0.24, [0.14, 0.33], *p* <.001).

### Multilevel Models

In fitting the means-only model of momentary emotion dysregulation, we derived an intra-class correlation (ICC) for our momentary emotion regulation difficulties measure, which specifies the respective variance explained at the between (= ICC) and within-person level (= 1-ICC). The ICC derived for emotion dysregulation was estimated to be 0.5014, indicating that 50.14% of the variability in participant emotion dysregulation across 28 EMA measurements was explained by between-person differences in dysregulation, while 49.86% of the variability in momentary emotion dysregulation was explained by the within-person fluctuations in dysregulation across the one-week period of assessments.

Next, we added momentary between- and within-person negative affect as fixed effect predictors of levels of emotion dysregulation. In this fixed-effects framework, the between-person fixed effect represents the person-mean scores in negative affect across the longitudinal period. The fixed effect of within-person variability represents the *average* level of intra-individual variability across the entire sample. Analyses revealed that both between-person negative affect (*β* = 0.97, [0.85, 1.10], *p* <.001) and within-person negative affect (*β* = 0.59, [0.56, 0.61], *p* <.001) significantly predicted increased momentary emotion dysregulation, controlling for age, sex, race/ethnicity, and the effects of the opposite predictor. Thus, higher average levels of negative affect were significantly associated with increased emotion dysregulation. Similarly, higher average within-person variability in negative affect within the sample also predicted increased levels of emotion dysregulation. This model demonstrated superior fit to the means-only model (*p* <.001).

Third, we added within-person negative affect as a random effect to test the degree to which intra-individual variability in negative affect predicted *variability* in emotion dysregulation. Fixed effect predictors representing between-person differences (*β* = 0.89, [0.78, 1.00], *p* <.001) and average within-person variability (*β* = 0.54, [0.46, 0.61], *p* <.001) in negative affect remained significant predictors of emotion dysregulation levels. The random effect for within-person variability in negative affect also significantly predicted variability in emotion dysregulation (*β* = 0.37, [0.32, 0.43]), controlling for covariates and the effects of the fixed effect predictors. Thus, greater intra-individual variability in negative affect was associated with greater fluctuations in emotion dysregulation. This model, which included the random effect of within-person negative affect, showed superior model fit compared to previous model (*p* <.001), which only included fixed effect predictors.

### Moderation of Negative Affect Associations

We evaluated cross-level moderation effects by determining whether the associations between within-person negative affect and emotion dysregulation varied based upon levels of maladaptive personality traits. After adding each personality trait individually the mixed effects model, we found no significant evidence of moderation (all *p*’s > 0.085), while all effects of between-person and within-person negative affect remained significant (all *p*’s < 0.001).

Finally, we examined whether between-person levels of negative affect moderated the association between within-person negative affect and overall momentary levels emotion dysregulation. The interaction term was significant (*β* = 0.06, [0.02, 0.09], *p* =.002), indicating that the positive association between within-person variability and momentary levels of emotion dysregulation was stronger for individuals with higher between-person levels of negative affect (see Fig. 1). Notably, the fixed effect of between-person negative affect (*β* = 0.97, [0.85, 1.09], *p* <.001) and the random effect of within-person negative affect (*β* = 0.36, [0.31, 0.42]) both remained significant, while the fixed effect of within-person negative affect became non-significant (*p* =.363). The inclusion of this moderation effect resulted in a significant improvement in fit compared to the previous model (*p* =.003), which included the random effect of within-person negative affect but not the cross-level moderation effects.

## Discussion

The present study evaluated the unique associations between maladaptive personality traits, negative affect, and emotion dysregulation among college students in both cross-sectional and intensive longitudinal contexts. Our findings revealed notable differences among maladaptive personality traits as predictors of dysregulation and identified negative affect as a key predictor of both overall levels and variability in emotion dysregulation at a momentary level. Below, we discuss our findings in detail and expound upon several implications regarding the empirical and clinical assessment of dysregulation.

### Differences Across Personality Trait Predictors

Overall, our hypotheses regarding unique associations between maladaptive personality traits and dimensions of emotion dysregulation were largely supported by our findings. Negative affectivity stood out as the strongest trait predictor of dysregulation, while psychoticism emerged an appreciably stronger predictor than expected. In contrast to prior work (Pollock et al., 2016), we found no unique effects of antagonism and stronger and more widespread effects of psychoticism on emotion dysregulation dimensions. These differences may be due to our over-selection for symptoms of ADHD and depression for the EMA period. Given that both ADHD (Bunford et al., 2015; Martel, 2009) and depression (Joormann & Gotlib, 2010) are associated with poorer emotion regulation, increased severity of those symptoms in our sample may have contributed to our relatively larger effect sizes. Increased clinical severity may have also increased our power to detect the effects of trait psychoticism, in particular, compared to studies in which floor effects may mask its effect due to skewness in non-clinical samples (Bach et al., 2018). Although measurement used in the current study precludes an evaluation of the role of antagonism in adaptive emotion regulation, the absence of unique associations between antagonism and dysregulation may indicate that trait antagonism is less reliably related to dysregulation or that its relative influence on dysregulation is less salient when compared to other traits like negative affectivity and psychoticism, particularly in samples with elevated psychopathology symptoms.

### Multilevel Effects on Momentary Dysregulation

Multilevel models revealed substantial within-person variability in momentary dysregulation, consistent with prior work (McMahon & Naragon-Gainey, 2019). Consistent with our hypotheses, between-person and within-person fixed effects of momentary negative affect each significantly predicted greater momentary levels of dysregulation, and within-person variability in negative affect predicted greater variability in dysregulation. The present findings, in conjunction with prior work, strongly suggest that the emotion regulation process is influenced by both trait differences between individuals (Hughes et al., 2020) and by situation or context dependent factors (Wilms et al., 2020), such as perceived control (Haines et al., 2016) or type of emotion (Smith et al., 2023). The two-fold influence on emotion regulation by trait and state level predictors is a theme reflected in the present work through the separation of negative affect into its between- and within-person subcomponents. Results showing that both overall levels of and momentary fluctuations in negative affect contribute to emotion dysregulation are in line with several past studies predicting regulation strategy usage (Brans et al., 2013; Smith et al., 2023; Wenzel et al., 2022).

### Trait Moderators of Negative Affect

We did not find that trait factors significantly moderated the impact of negative affect on momentary dysregulation. This stands in contrast to recent work, which found that trait factors (i.e., negative urgency and distress tolerance) moderated the impact of negative affect variability on emotion disengagement strategies (e.g., avoidance, suppression; Sandel-Fernandez et al., 2023). The lack of this moderation effect in the present study may be because we focused on trait variables with high endurance (i.e., personality domains; see Damian et al., 2019), which may be too stable to meaningfully affect the dynamic variability observed in momentary dysregulation. By contrast, “trait-like tendencies” (Sandel-Fernandez et al., 2023) with lower endurance, like negative urgency and distress tolerance, may be more likely to modulate the effects of momentary affect and consequently impact regulation.

On the other hand, between-person levels of negative affect did emerge as a moderator in this study, such that the association between negative affect variability and levels of momentary dysregulation was stronger for individuals with higher overall levels of momentary negative affect. Interactions between trait and state-levels of the same construct have been investigated before, such as in Endler and Hunt’s (1968) interaction model of anxiety, which found that the interaction between trait levels of anxiety and situational responses accounted for more variance than either of the main effects alone (Endler & Hunt, 1968). These findings demonstrated that a more precise understanding of anxiety was found by analyzing trait differences as they played out across varying situational contexts. Likewise, the present findings may suggest that, as one’s baseline affect worsens, one’s ability to regulate emotions in response to situational variability in negative affect becomes increasingly impaired.

### Implications

Our findings underscore the value of increasing efforts to evaluate emotion regulation across momentary timescales (Colombo et al., 2020). Momentary fluctuations in affect and other psychological and physiological processes appear to be highly relevant mechanisms for influencing emotion regulation (Sandel-Fernandez et al., 2023; Thompson et al., 2024). While the present study only investigated negative affect, numerous situational determinants influence emotion and one’s response in real moment-to-moment experiences, such as positive affect (Smith et al., 2023), interpersonal and achievement events (Pollock et al., 2016), and one’s goals for employing emotion regulation (English et al., 2017). Therefore, an analysis of several momentary predictors of dysregulation together may be a fruitful pursuit for comprehensively understanding the mechanisms that facilitate its progression to and maintenance of psychopathology.

Due to its transdiagnostic effects, emotion dysregulation has been identified as a malleable target for intervention in multiple modalities of treatment (Cameron & Jago, 2008; Eadeh et al., 2021; Quoidbach et al., 2015), and the identification of its momentary antecedents may help to reveal etiological mechanisms of psychiatric disorders like depression and anxiety (D’Avanzato et al., 2013) and BPD (Daros et al., 2018). Our findings suggest that trait predictors of dysregulation may be most useful when calculating risk and making predictions between groups, whereas state-level predictors of dysregulation may be more malleable in clinical settings and useful, in conjunction with other proximal antecedents, for signaling the delivery of real-time interventions (see Coppersmith et al., 2022).

### Limitations

The findings of these analyses should be considered in the context of their limitations. First, this study recruited a college student sample, and, while over-selected for higher levels of psychopathology, participants were not clinically diagnosed, which limits the degree to which these findings may be generalized to clinical populations. Second, future research may benefit from using longer variations of the PID-5 (Krueger et al., 2012; Maples et al., 2015) to assess facet-level information for more fine-grained features of personality pathology (e.g., specific features of negative affectivity, such as emotional lability, anxiousness, and separation insecurity). Additionally, while the PID-5 may help to better capture dimensionality in personality pathology, lack of measurement invariance across White and Black Americans (Bagby et al., 2022) may limit its utility as a measure and impact generalizability of findings among diverse populations. Future research should utilize other dimensional measures of personality pathology (e.g., Preliminary Scales for ICD-11 Personality Disorder; Clark et al., 2021) and measures of emotion dysregulation specifically designed for momentary designs (McMahon & Naragon-Gainey, 2020; Medland et al., 2020) to specify which traits exert most influence on regulation and determine how regulation processes vary uniquely in real-time contexts.

## Conclusion

Overall, the current study demonstrated that multiple maladaptive personality traits, particularly negative affectivity, uniquely contribute to several dimensions of emotion dysregulation among college students. When evaluating momentary variability in emotion dysregulation across a one-week EMA period, between- and within-person differences in negative affect predicted increased levels and variability of emotion dysregulation. Findings support the continued use of ambulatory assessment methodologies for evaluating emotion regulation to clarify its role in relation to psychopathology and to aid in the creation and delivery of real-time interventions.

## Electronic Supplementary Material

Below is the link to the electronic supplementary material.

Supplementary Material 1
