# Supplementary material for: Emotion Dysregulation in College Students: Contributions of Maladaptive Personality Traits and Momentary Affect
Source: J Psychopathol Behav Assess. 2025 Aug 4;47(3):65. doi: 10.1007/s10862-025-10243-7 (PMC12322088; doi:10.1007/s10862-025-10243-7)
Supplement: Supplementary file 1 — Supplementary Material 1 [file 10862_2025_10243_MOESM1_ESM.docx]

**SUPPLEMENTARY MATERIALS**

**APPENDIX A: Other Measures Collected**

In addition to the measures reported in this study, participants completed the following measures as part of the study:

**Lab Visit or Online Initial Questionnaires**

Barkley Adult ADHD Rating-Scale-IV, Current and Childhood Symptoms

Barkley Deficit in Executive Function Scale

Barkley Functional Impairment Scale

Externalizing Spectrum Inventory

Monetary Choice Questionnaire

Perth Emotion Reactivity Scale

Difficulties in Emotion Regulation Scale

Positive and Negative Affect Scale

Inventory of Depression and Anxiety Symptoms-II

Social Media Use Questionnaire

ASEBA Adult Self-Report

**Cognitive Tasks (completed by in-person participants and 55% of online participants)**

Stop Signal Reaction Time

Letter Memory Test

Balloon Analogue Risk Task

Delay Discounting Task

Delis-Kaplan Color Word Interference Task

**Ecological Momentary Assessment Measures**

ADHD behaviors (4 items)

Depression symptoms (2 items)

Minutes on social media websites (reported from phone metadata)

Negative Social Comparison Ratings (2 items)

Fear of Missing Out Scale – (2 items)

Bedtime and Waking time from prior day (morning survey only)

Number of exercise minutes from prior day (morning survey only)

Number of alcoholic drinks from prior day (morning survey only)
